# Postoperative human motion evaluation in orthopedics based on kinematic constraints and improved IMU-generated data processing algorithm

**DOI:** 10.1371/journal.pone.0323821

**Published:** 2025-05-22

**Authors:** Linzhi Xie, Zhengnan Li

**Affiliations:** 1 Department of Physical Education, Ganzhou Teachers College, Ganzhou, China; 2 Department of Sports Medicine, Ganzhou People’s Hospital, Ganzhou, China.; Ningbo University, CHINA

## Abstract

Traditional methods for evaluating postoperative human motion in orthopedics suffer from high equipment costs, time-consuming detection processes, and can only provide static two-dimensional image information. To improve the efficiency and accuracy of postoperative human motion assessment in orthopedics, this study proposes an improved algorithm on the basis of kinematic constraints and inertial measurement units for postoperative human motion assessment in orthopedics. Compared with other methods, the average error and standard deviation of measuring human joint range of motion after orthopedic surgery based on inertial measurement units were the smallest. The average error and standard deviation of measuring human wrist joint range of motion were 1.23° and 1.63°, respectively. The efficiency and accuracy of measuring human joint range of motion after orthopedic surgery based on inertial measurement units were the highest. The efficiency and accuracy were 4s and 98%, respectively. The accuracy of calculating the human joint Angle after orthopedic surgery based on kinematic constraints is also better than other traditional algorithms. The method has high accuracy and applicability, which can effectively improve the monitoring efficiency of postoperative human motion assessment in orthopedics, reduce the error of postoperative human motion assessment in orthopedics, and effectively support personalized management and intervention, further providing reference for the optimization of postoperative human motion assessment in orthopedics.

## 1. Introduction

With the improvement of living standards, people are increasingly focusing on pursuing a healthy lifestyle and are willing to spend more time on fitness activities. Active and scientific fitness has become a conscious choice for more people [1]. In recent years, some people have blindly continued high-intensity exercise without timely supplementation of sufficient nutrition, resulting in a decrease in the body’s self-repair ability, the impact of exercise, and long-term damage to bones leading to fractures [2]. Therefore, it is very important to detect and evaluate the bone damage repair after human exercise.

However, there are still many problems in the current methods used to evaluate human movement after orthopedic surgery. Traditional methods often face high equipment costs, time-consuming detection processes, and limitations in providing static two-dimensional image information. These problems seriously affect the efficiency and accuracy of orthopedic postoperative motion evaluation. In addition, existing detection devices are inconvenient to wear, affecting patients’ normal movement and comfort, limiting their feasibility in long-term continuous monitoring. At the same time, the detection efficiency and accuracy of these methods are low, and it is difficult to meet the requirements of widespread application in clinical and daily environments [3,4].

To solve these problems, an Inertial Measurement Unit (IMU) algorithm based on kinematic constraints and inertial Measurement Unit (IMU) is proposed to evaluate human movement after orthopedic surgery. As a lightweight, low-power and small-size device, IMU can measure the three-axis attitude Angle and acceleration of objects in real time, and provide high-frequency motion state data. It is suitable for real-time control and does not depend on external signals, and can work in a signal-free environment [5,6]. However, IMU has a cumulative error problem in the measurement process, which may affect its measurement accuracy. Therefore, this study innovatively introduced the Global Positioning System (GPS) to improve the IMU, and calculated the joint Angle with kinematic constraints to solve the cumulative error of the IMU itself, so as to improve the measurement accuracy. The aim of this study is to provide strong support for individualized management and intervention of orthopaedic postoperative rehabilitation.

The main contribution of this study is to propose a postoperative human motion evaluation method for orthopedic surgery based on kinematic constraints and an improved IMU algorithm, in order to improve the efficiency and accuracy of the evaluation. Simultaneously innovatively combining GPS and IMU to correct the accumulated errors of IMU and improve the reliability of motion data. In addition, the use of kinematic constraint methods simplifies the sensor installation process, eliminating the need for manual calibration and distance measurement, making this method more clinically valuable.

## 2. Related works

IMU, as an important component of modern sensor technology, has become the first choice in many application fields due to its light weight, low power consumption, small size, and high reliability. It is used for real-time monitoring and calculation of acceleration, angular velocity, and attitude information of objects [7,8]. Many studies have focused on improving the accuracy and reliability of IMUs in joint Angle measurement through algorithm optimization, sensor fusion, or other technical means. For example V. B. Semwal aimed to address diverse joint motion patterns in different walking styles, which increased the recognition difficulty due to the significant differences in walking styles among individuals. The IMU sensor was used to identify different human joint patterns of different walking styles. The method accurately recognized the motion patterns of different human joints under different walking styles, with a recognition accuracy of 92% [9]. S. Y. Song et al. aimed to accurately estimate the relative angle between two adjacent joints in applications such as human motion analysis or robot pose estimation. A convenient IMU was proposed, which utilized two 6-axis IMUs to calculate the relative angle, each IMU containing an accelerometer and gyroscope. The method exhibited good performance under different motion modes and environmental conditions, with strong robustness and adaptability [10]. X. Zhang et al. aimed to accurately estimate human gait in lower limb exoskeletons. A novel method based on IMUs was proposed to assess the gait stages of pilots in continuous multi-motion modes. The gait mode classification accuracy using 5 IMUs was 98.58% [11]. Y. Gu et al. aimed to address the measurement errors in magnetic IMUs due to magnetic interference in indoor environments, and the low performance of flexible sensors in terms of linearity and accuracy. A three-stage real-time adaptive anti-interference data fusion strategy was built. The data fusion algorithm exhibited high precision performance in magnetic interference environments, with maximum root mean square errors of 1.23°, 1.15°, and 3.67° per axis for the measured continuous 3D motion angles of the knee joint, hip, and elbow crossing [12].

In order to overcome the accumulated error of a single sensor, environmental interference or insufficient measurement accuracy, many studies have adopted sensor fusion technology, combined with a variety of sensor data or optimization algorithms, to improve the accuracy and reliability of joint Angle measurement. For example, D. Xu studied the risk of anterior cruciate ligament (ACL) injury when landing on one leg after fatigue and found that changes in ankle movement patterns were associated with an increased risk of ACL injury. By collecting data from 56 subjects, the relationship between initial contact Angle (AIC), range of motion (AROM) and ACL peak force (PAF) of ankle joint was analyzed, and the ACL force prediction model based on deep learning was established. The results show that AIC and AROM are significantly correlated with PAF, and the model has excellent predictive performance [13]. X. Chen et al. improved the sensitivity, detection range, and stability of flexible pressure sensors in human joint motion monitoring. A flexible pressure sensor was designed. The sensor designed in the study had excellent sensitivity, wide measurement range, fast response, and excellent repeatability. In addition, pressure sensors had high accuracy in detecting and analyzing human finger bending and elbow bending motions [14]. X. Huang et al. aimed to improve the sensitivity of motion detection. A scheme for waveguide strain sensors in motion detection of human joints and muscles was proposed. The sensitivity was 3.9 times and 1.2 times higher than that of sensors made of pure polydimethylsiloxane and polymethyl methacrylate. It had a high signal-to-noise ratio [15].

Human joint motion monitoring is of crucial importance in orthopedic surgery, as it can provide doctors with real-time and objective data on joint function recovery after surgery, helping to accurately evaluate surgical outcomes and guide rehabilitation training [16,17]. With the rapid development of machine learning and deep learning techniques, these methods have been widely used in human motion monitoring and joint Angle measurement. For example, B. S. Lin et al. aimed to address the challenges faced by motion trajectory tracking systems on the basis of IMUs, like low adaptability to various activities and limited ability to perform two-dimensional trajectory estimation. A residual neural network was proposed to estimate the daily activity trajectory of users. The method accurately predicted the motion trajectory of daily activities, with a relative trajectory error of 0.81m for walking [18]. D. Xu et al. proposed a gait feature selection method based on meta-heuristic optimization, aiming at identifying key gait features to improve the accuracy and efficiency of gait pattern recognition. In this study, 36 features were extracted from 800 sets of gait data, and 10 optimal features were selected by optimization algorithm, including 6 ankle joint correlation and 4 knee joint correlation. Finally, the recognition accuracy of 99.81% is achieved, which is significantly better than the traditional method [19]. P. Xu et al. aimed to solve high installation requirements and complex operation in existing compensation motion mode detection methods. A torso restraint and compensation motion mode detection system was designed. The method exhibited good performance, with an F1 score of 0.9256 for trunk forward tilt detection, 0.9190 for scapular elevation detection, and 0.826 for trunk rotation detection [20].

In summary, although there are a variety of joint Angle measurement methods based on IMU, there are still some shortcomings. For example, some studies have achieved high-precision joint Angle measurements, but rely on complex sensor installation and calibration processes, limiting their widespread use in clinical Settings. In addition, although some studies have improved the measurement accuracy through deep learning or machine learning, they require high computing resources and are difficult to achieve real-time monitoring. Progress has been made in solving the cumulative error problem of IMU, but the potential effect of kinematic constraints on improving measurement accuracy has not been fully considered. This study systematically optimized and innovated the limitations of existing orthopedic postoperative motion evaluation techniques, and introduced kinematic constraints to solve the problem of IMU accumulated error, while simplifying the sensor installation process without manual calibration and distance measurement. In addition, the IMU is improved in combination with GPS to further improve the reliability and measurement accuracy of motion data.

## 3. Postoperative human motion assessment in orthopedics

### 3.1. Measurement of human joint range of motion after orthopedic surgery based on IMU

**Fig 1:**
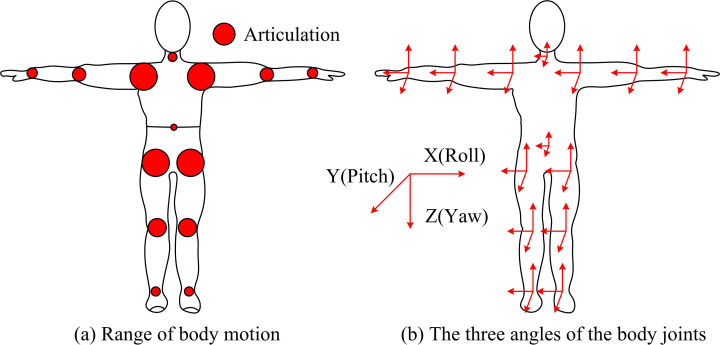
Three angles of body motion range and joints.

Joint range of motion signifies the maximum curvature that can be achieved during joint motion. It is a indicator for evaluating the range and degree of joint motion function damage [21,22]. Due to the fact that orthopedic surgery often involves the repair or reconstruction of bones, joints, and surrounding soft tissues, changes in joint mobility after surgery directly reflect the surgical outcome and progress of rehabilitation treatment. IMU can measure the three-dimensional motion of joints in real time and continuously, providing dynamic motion information without the need for complex equipment settings or dedicated space, and is easy to operate [23,24]. Therefore, the IMU is used to measure the motion of human joints. Range of Motion (ROM) is the maximum Angle of motion that a knuckle can achieve during movement. It is an important indicator of joint function and movement ability, reflecting the flexibility and health of the joint. In orthopedic postoperative rehabilitation, ROM is a key parameter to measure the effect of surgery and the progress of rehabilitation. In three-dimensional space, the motion of the joint can usually be described by three angles, which correspond to the rotation of the joint in different axes. Pitch: The Angle of rotation of the joint around the front and back axes, such as flexion and extension of the knee joint. Roll Angle (Roll): The Angle of rotation of the joint around the left and right axis, such as the inversion and inversion of the ankle joint. Yaw: The Angle of rotation of a joint about its vertical axis, such as internal and external rotation of the hip joint. When modeling the human skeleton, the study focused on the movement of key body parts and joints to balance the complexity and realism of the model. The range of motion of the body and the three angles of the joints are shown in Fig 1.

**Fig 2:**
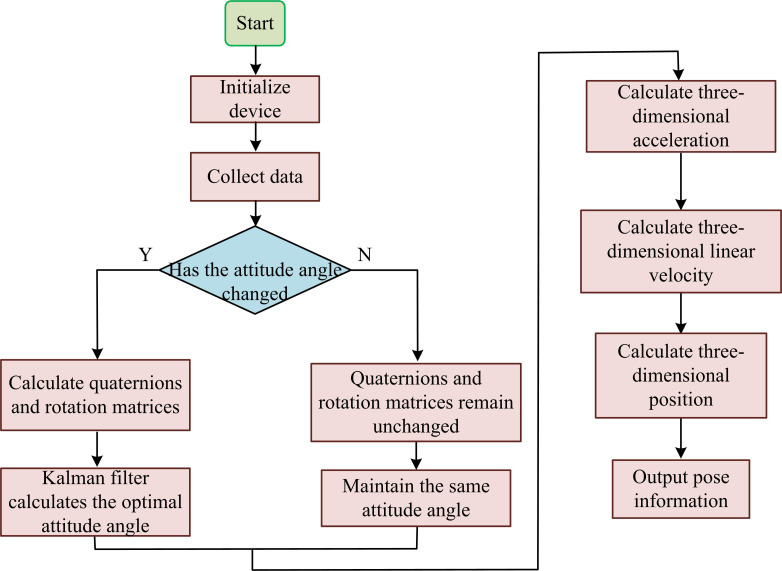
Steps for measuring human joint range of motion after orthopedic surgery based on IMU.

The IMU is an integrated sensor consisting of a three-axis accelerometer and a three-axis gyroscope. The accelerometer is used to measure the acceleration of an object in three directions, the attitude can be determined by the acceleration of gravity (1g) at rest, and the Z-axis reading is 1g when placed horizontally, and the X-axis and Y-axis reading are 0. The gyroscope measures the angular speed of the object around three axes, and the Angle of rotation can be calculated by integrating, such as the angular speed of 10°/s around the Z axis, and the Angle of rotation is 10° after 1 second. Due to the advantages and disadvantages of accelerometers and gyroscopes, data fusion is usually needed to improve the measurement accuracy. The common methods include Kalman filtering and complementary filtering. However, due to the cumulative error problem of IMU itself in measurement, GPS’s absolute positioning capability can correct the cumulative error. Therefore, the GPS is used to improve the IMU, as follows. First, the raw data of IMU and GPS are collected, where IMU provides acceleration and angular velocity information, while GPS provides position and velocity information. Secondly, the state estimation and covariance matrix of the Kalman filter are initialized, and the IMU data and system model are used to predict the current state. Finally, GPS data is used to correct the predicted state, thereby improving the accuracy of position and velocity estimation. The steps for measuring human joint range of motion after orthopedic surgery based on IMU improved algorithm are shown in Fig 2.

In Fig 2, the steps for measuring human joint range of motion based on IMU are as follows. Firstly, the device is initialized. Secondly, multiple sets of data are obtained through three-axis accelerometers and three-axis gyroscopes. When the attitude angle changes, the system will calculate quaternions and rotation matrices, and optimize the calculation of attitude angle through Kalman filtering algorithm. The implementation steps of Kalman filter are as follows: initialize state vector $x_{0}$ , covariance matrix $P_{0}$, process noise covariance matrix Q and measurement noise covariance matrix R. The state equation and the optimal state estimate of the previous moment are used to predict the state and covariance of the current moment. For the specific process, see Equation (1).


$$\left\{ \begin{array}{c} {}*35lx_{k|k-1}=F_{k}x_{k-1|k-1}+B_{k}u_{k}\\ P_{k|k-1}=F_{k}P_{k-1|k-1}F_{k}^{T}+Q_{k} \end{array}\right.$$ (1)

In formula (1), $F_{k}$ represents the state transition matrix. $B_{k}$ is the control input matrix. $u_{k}$ is the control input. $Q_{k}$ is the process noise covariance matrix. The Kalman gain is calculated using the measurement equation, the current moment measurements, and the predicted state values, and the state estimates and covariances are updated. The process is shown in Equation (2).


$$\left\{ \begin{array}{c} {}*35lK_{k}=P_{k|k-1}H_{k}^{T}{\left(H_{k}P_{k|k-1}H_{k}^{T}+R_{k}\right)}^{-1}\\ x_{k|k}=x_{k|k-1}+K_{k}\left(z_{k}-H_{k}x_{k|k-1}\right)\\ P_{k|k}=\left(I-K_{k}H_{k}\right)P_{k|k-1} \end{array}\right.$$ (2)

In formula (2), $H_{k}$ is the observation matrix, $R_{k}$ is the measurement noise covariance matrix, $z_{k}$ is the measurement value, $K_{k}$ is the Kalman gain. For IMU data, Q can be set to a small value because of its high measurement frequency and stable data. A small R can reduce the impact of measurement noise on the filtering results, but too small may lead to over-reliance on the measurement data and neglect of the system model. For GPS data, R can be set to a larger value because of its low measurement frequency and large error. The initial P should be set reasonably to reflect the uncertainty of the state estimation and ensure a reasonable convergence rate of the filter. Improper P may result in slow convergence or divergence of the filter. The initial state vector x 0 can be set to the first measurement or estimated based on prior knowledge. The initial covariance matrix $P_{0}$ is usually set to the identity matrix, or adjusted for uncertainty about the initial state. The Settings of F and H depend on the specific system model and measurement model. For example, for the fusion of IMU and GPS data, F can be expressed as formula (3).


$$F=\left[\begin{array}{ccc} 1 & \Delta t & 0\\ 0 & 1 & \Delta t\\ 0 & 0 & 1 \end{array}\right]$$ (3)

The calculation of the Kalman gain $K_{k}$ determines the degree of reliance of the filter on the measured data. A larger $K_{k}$ value means that the filter trusts the measurement data more, while a smaller $K_{k}$ value means that the filter trusts the model prediction more. By adjusting the values of Q and R, the influence of measurement noise and model error can be effectively reduced, and the accuracy of attitude estimation can be improved. Finally, the acceleration and linear velocity in the coordinate system are calculated and the pose information is output.

When collecting data with a three-axis accelerometer and a three-axis gyroscope, ensuring accuracy and handling noise are key. To this end, the research first eliminates zero bias, scale factor error and cross-coupling error by initializing and calibrating the sensor to ensure the measurement accuracy. Secondly, Kalman filter is used to fuse accelerometer and gyroscope data, and GPS is used to correct IMU accumulated errors to improve data reliability. In addition, the joint Angle is calculated based on the kinematic constraint model to reduce the influence of sensor installation errors and soft tissue interference. In terms of noise processing, low-pass filtering effectively removes high-frequency noise by setting the cutoff frequency, retains the low-frequency motion signal, and reduces short-term fluctuation interference. Data preprocessing includes outlier removal, data smoothing and normalization to improve data quality. Wavelet transform further decomposed the signal, removed the noise component by threshold processing, and then reconstructed the signal to extract the effective information. The kinematic constraint model combines physical laws to correct sensor data and reduce installation errors and soft tissue interference.

**Fig 3:**
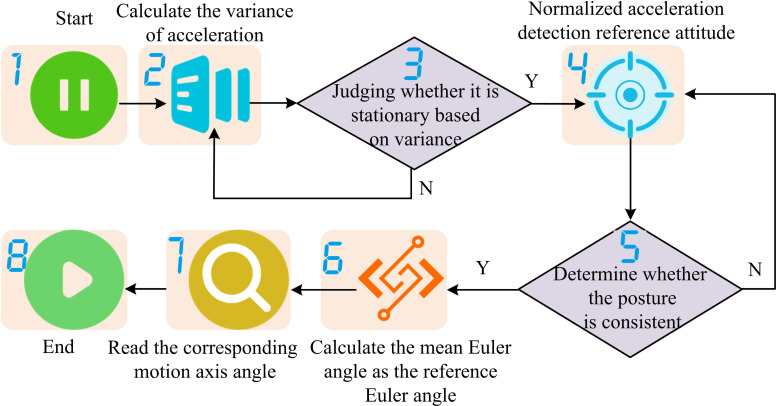
Step diagram for detecting the independent three-axis posture of human joints in the carrier coordinate system based on IMU accelerometer.

In the case of fast movement or vibration, the key to ensuring measurement accuracy is to optimize data processing and sensor configuration. First, the IMU device with a high sampling rate ensures that high-frequency motion information can be captured. Secondly, combined with Kalman filter or extended Kalman filter algorithm, the cumulative error of accelerometer and gyroscope is corrected in real time, and the influence of soft tissue interference and sensor installation error is reduced by kinematic constraint model. In addition, the signal is analyzed on a multi-scale by wavelet transformation to remove high-frequency noise while preserving key features of motion. The accelerometer of IMU can determine the posture based on the acceleration in three axis directions. The steps of detecting the independent three-axis posture of human joints in the carrier coordinate system based on IMU accelerometer are shown in Fig 3.

**Fig 4:**
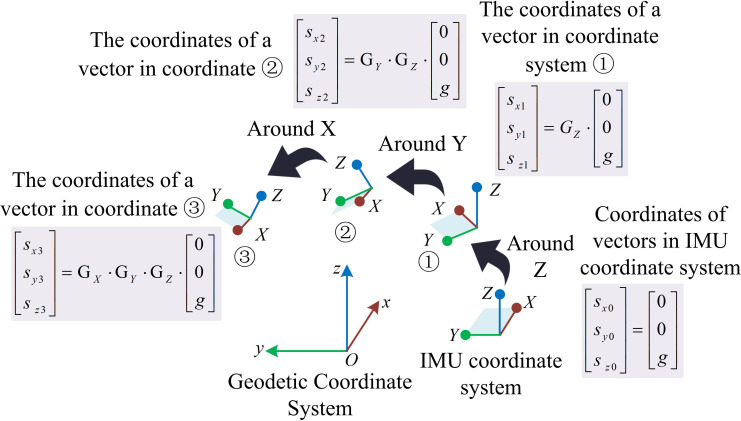
The decomposition process of IMU rotating three times in the order of ZYX.

As shown in Fig 3, the steps for detecting independent three-axis acceleration of human joints in the carrier coordinate system based on IMU accelerometer are as follows. Firstly, the IMU initializes and starts collecting data to calculate the variance of acceleration generated by the limb during the motion process within a specific detection time. Secondly, whether the human body is in a stationary state is determined based on the calculated variance of acceleration. If not in a stationary state, the variance of acceleration generated by the limb during the motion process within a specific detection time is recalculated. If in a stationary state, the acceleration data is normalized to determine the reference attitude and compare it with the current attitude. After confirming the posture, the system will determine the reference Euler angle by calculating the average Euler angle during the stationary period in the order of ZYX, and then calculate the motion angle. When the accelerometer is placed horizontally, that is, when the Z-axis is vertically upward, the Z-axis can read 1g, and the values in the X and Y-axis are 0, signified as ${\left[0,0,g\right]}^{T}$. When the accelerometer rotates in a certain posture, the gravitational acceleration will generate corresponding components on the three axes. At this time, the three values are the coordinates in the new coordinate system of vector ${\left[0,0,g\right]}^{T}$. The decomposition process of rotating the IMU three times in the order of ZYX is shown in Fig 4.

In Fig 4, the decomposition process of rotating the IMU three times in the order of ZYX is as follows. Firstly, the coordinates of vector ${\left[0,0,g\right]}^{T}$ in the initial attitude IMU coordinate system are transformed into ① through a rotation matrix $G_{Z}$ around the Z-axis. The vector coordinates at this point are shown in Equation (4).


$$\begin{array}{c} {}[\begin{array}{c} s_{x1}\\ s_{y1}\\ sz1 \end{array}]=G_{Z}\cdot \left[\begin{array}{c} 0\\ 0\\ g \end{array}\right] \end{array}$$ (4)

In Equation (4), ${\left[s_{x1},s_{y1},s_{z1}\right]}^{T}$ represents the coordinates of ${\left[0,0,g\right]}^{T}$ in ①. Next, the vectors in ① are transformed into coordinate system ② through a rotation matrix $G_{Y}$ around the Y-axis. The vector coordinates at this point are shown in Equation (5).


$$\begin{array}{c} {}[\begin{array}{c} s_{x2}\\ s_{y2}\\ sz2 \end{array}]=G_{Y}\cdot G_{Z}\cdot \left[\begin{array}{c} 0\\ 0\\ g \end{array}\right] \end{array}$$ (5)

In Equation (5), ${\left[s_{x2},s_{y2},s_{z2}\right]}^{T}$ represents the coordinates of ${\left[0,0,g\right]}^{T}$ in ②. Finally, the vectors in ② are transformed into ③ through a rotation matrix $G_{X}$ around the X-axis. The coordinates of vector ${\left[0,0,g\right]}^{T}$ in ③ are the values read by the accelerometer.


$$\begin{array}{c} {}[\begin{array}{c} s_{x3}\\ s_{y3}\\ sz3 \end{array}]=G_{X}\cdot G_{Y}\cdot G_{Z}\cdot \left[\begin{array}{c} 0\\ 0\\ g \end{array}\right] \end{array}$$ (6)

In Equation (6), ${\left[s_{x3},s_{y3},s_{z3}\right]}^{T}$ represents the coordinates of ${\left[0,0,g\right]}^{T}$ in ③. Due to the constant gravitational acceleration detected by the accelerometer during rotation around the Z-axis, it is not possible to calculate the heading angle through the accelerometer. At this time, the pitch and roll angles for rotation around the Y and X axes are calculated using Equation (7).


$$\left\{ \begin{array}{c} roll_{1}=\arctan\left(\frac{s_{y3}}{s_{z3}}\right)\\ pitch_{1}=-\arctan\left(\frac{s_{x3}}{\sqrt{s_{y3}^{2}+s_{z3}^{2}}}\right) \end{array}\right.$$ (7)

**Fig 5:**
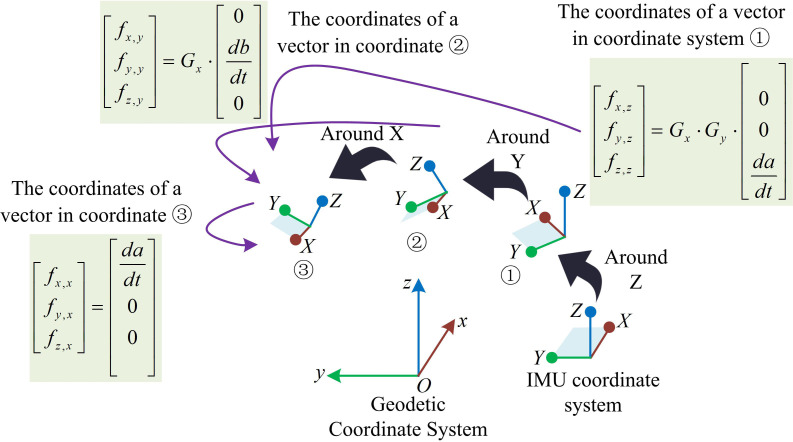
Shows the step diagram of decomposing the attitude angular velocity into 3 rotations in the order of ZYX.

In Equation (7), roll1 represents the roll angle around X. $pitch_{1}$ represents the pitch angle around Y. The gyroscope of IMU measures the angular velocity rotating around three axes, so the angle can be obtained by integrating the angular velocity. Assuming that at the n-th moment, the attitude angle of the IMU is a, b, and c. This indicates that the IMU coordinate system has reached its current posture by rotating a, b, and c angles around the Z, Y, and X axes, respectively, from its initial position. At the next time n + 1, the attitude angles become a + Δa, b + Δb, and c + Δc, and this attitude also undergoes three rotation processes. The steps of decomposing the attitude angular velocity into 3 rotations in ZYX order are shown in Fig 5.

As shown in Fig 5, the decomposition of attitude angular velocity is as follows. Firstly, in the IMU coordinate system, the angular velocity of the heading angle around the Z-axis is represented by the unit vector ${\left[0,0,1\right]}^{T}$. The first rotation is around the Z. ${\left[0,0,dc/dt\;\right]}^{T}$ signifies the angular velocity around the Z in coordinate system ①. Secondly, after the IMU rotates around the Y-axis and X-axis, the original angular velocity vector also needs to be rotated twice through the corresponding rotation matrix to obtain the correct representation in the new coordinate system. Finally, all transformed angular velocity components are added to obtain the data read by the gyroscope in coordinate system ③, which includes angular velocity components around the Z, Y, and X. The data ${\left[f_{x},f_{y},f_{z}\right]}^{T}$ read by the gyroscope in coordinate system ③ is shown in Equation (8).


$$\begin{array}{c} {}[\begin{array}{c} f_{x}\\ f_{y}\\ f_{z} \end{array}]=G_{X}\cdot G_{Y}\cdot \left[\begin{array}{c} 0\\ 0\\ \frac{da}{dt} \end{array}\right]+G_{X}\cdot \left[\begin{array}{c} 0\\ \frac{db}{dt}\\ 0 \end{array}\right]+\left[\begin{array}{c} \frac{dc}{dt}\\ 0\\ 0 \end{array}\right] \end{array}$$ (8)

In Equation (8), ${\left[f_{x},f_{y},f_{z}\right]}^{T}$ represents the data read by the gyroscope in coordinate system ③. $\frac{dc}{dt}$ represents the angular velocity component around the Z. $\frac{db}{dt}$ represents the angular velocity component around the Y-axis. $\frac{da}{dt}$ represents the angular velocity component around the X. Assuming that the IMU state in coordinate system ③ is considered as the n-th time state, the angular velocity and other information required for position update can be inferred from the data read from the gyroscope, thereby achieving attitude update from the n-th time to the (n + 1)-th time. The angular velocity calculation required for position update is shown in Equation (9).


$$\begin{array}{c} \left[\begin{array}{c} \frac{dc}{dt}\\ \frac{db}{dt}\\ \frac{da}{dt} \end{array}\right]=\left[\begin{array}{ccc} 1 & \frac{\sin b\cdot \sin c}{\cos b} & \frac{\cos c\cdot \sin b}{\cos b}\\ 0 & \cos c & -\sin c\\ 0 & \frac{\sin c}{\cos b} & \frac{\sin c}{\cos b} \end{array}\right]\cdot \left[\begin{array}{c} f_{x}\\ f_{y}\\ f_{z} \end{array}\right] \end{array}$$ (9)

From the above analysis, the accelerometer can calculate the roll angle and dive angle on the basis of the perceived gravitational acceleration at rest, and the angle calculation is only related to the current posture. The gyroscope integrates the angular velocity within a time interval to obtain the angle transformation for each time, accumulates it to the previous attitude angle, and obtains a new attitude angle. The gyroscope can calculate three angles: roll angle, dive angle, and heading angle. The accelerometer can obtain a more accurate attitude at rest, while the gyroscope is sensitive to attitude changes during rotation. However, after continuous time integration, the error will continue to increase. Therefore, it is necessary to perform complementary fusion of gyroscope and accelerometer for attitude calculation. The roll angle and dive angle calculated by the accelerometer are fused with the roll angle, dive angle, and heading angle calculated by the gyroscope, as shown in Equation (10).


$$\left\{ \begin{array}{c} roll=roll_{2}+(roll_{1}-roll_{2}cdotK\\ pitch=pitch_{2}+\left(pitch_{1}-pitch_{2}\right)\cdot K\\ yaw=yaw_{2} \end{array}\right.$$ (10)

In Equation (10), K represents the proportionality coefficient. roll, pitch, and yaw respectively signify the roll angle, dive angle, and heading angle obtained by fusion. $roll_{1}$ and $roll_{1}$ respectively signify the roll angle and dive angle calculated by the accelerometer. $roll_{1}$, $pitch_{2}$ and $yaw_{2}$ respectively signify the roll angle, dive angle, and heading angle calculated by the gyroscope.

### 3.2. Calculation of human joint angles after orthopedic surgery based on kinematic constraints

**Fig 6:**
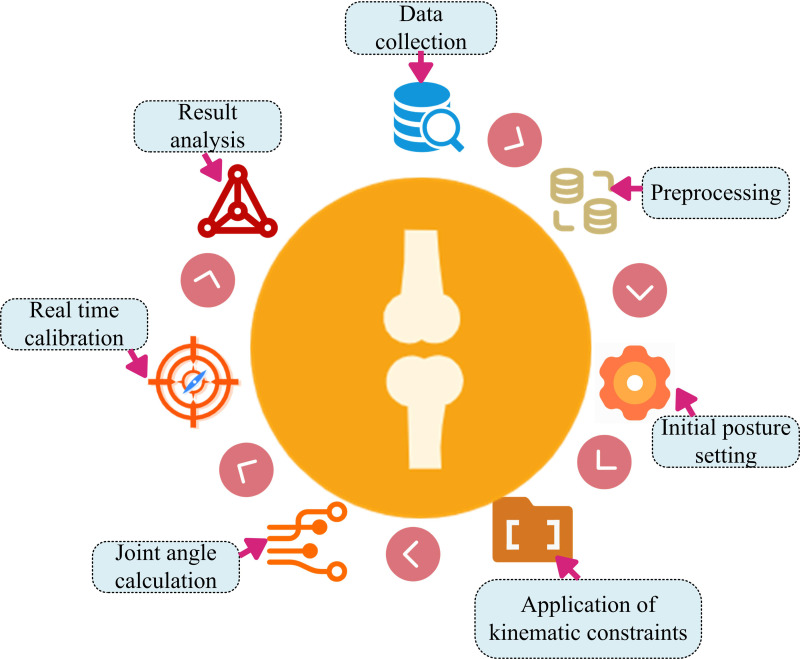
Steps for calculating human joint angles after orthopedic surgery based on kinematic constraints.

After measuring the range of motion of human joints after orthopedic surgery based on IMU, the joint angles after orthopedic surgery are calculated based on kinematic constraints. When using IMU for attitude estimation, it is crucial to correctly install sensors at the joints. To correctly install sensors to the joint area, manual calibration and distance measurement are required before measurement, which not only affects the patient’s experience, but also increases the operation complexity [25,26]. In contrast, the kinematic constraints can directly obtain the information of sensors in the local coordinate system during joint flexion and extension. This method does not require manual measurement or preset parameters, nor does it depend on whether the measured object can accurately perform specific poses or actions [27]. The kinematic constraint method not only simplifies the operation process, but also maintains measurement accuracy while improving user experience [28]. Therefore, the joint angles of the human body after orthopedic surgery are calculated based on kinematic constraints. and the calculation steps are shown in Fig 6.

**Fig 7:**
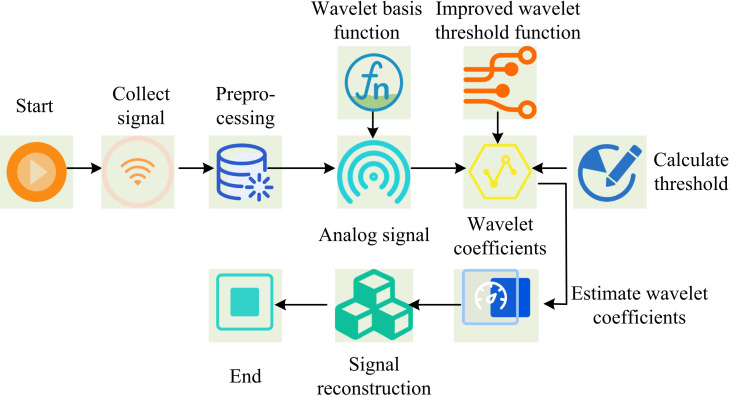
The denoising steps of human motion data based on wavelet transform.

In Fig 6, the steps for calculating the joint angles of the human body after orthopedic surgery based on kinematic constraints are as follows. Firstly, IMU sensors are used to collect motion data of the patient’s joints, and the collected motion data is preprocessed. Secondly, the initial position and orientation of the sensor on the body are determined. Finally, the joint angles are calculated based on kinematic constraints. IMU sensors may be affected by electronic or environmental noise during the measurement of patient joint motion data. Preprocessing steps can help identify and remove these noises, improving data accuracy. Wavelet transform is a new transform analysis method. The basic idea is to remove the Wavelet Decomposition Coefficients (WDC) corresponding to noise in different frequency bands based on the different intensity distribution characteristics, while retaining the WDC of the original signal. The processed coefficients are subjected to wavelet reconstruction to get a pure signal. It can analyze signals at different scales and positions, adapting to the local characteristics of signals [29,30]. Therefore, the study uses wavelet transform to denoise the motion data collected by IMU. The denoising steps are shown in Fig 7.

In Fig 7, the steps for denoising the motion data collected by IMU using wavelet transform are as follows. Firstly, the motion data collected by IMU is preprocessed. Secondly, using wavelet basis functions for wavelet decomposition, the signal is decomposed into wavelet coefficients at different scales. Signal characteristics, computational efficiency, symmetry and mathematical characteristics should be considered in the selection of wavelet function. The mutation signal is selected as Haar or low-order Daubechies wavelet. Smooth signals choose high order Daubechies or Symlet wavelets. Haar wavelet has simple structure and high computational efficiency. Symlet and Coiflet wavelet have good symmetry and can avoid edge effect. The selection also needs to consider orthogonality, tight support, etc., orthogonality ensures distortion free decomposition, and tight support is conducive to analyzing local characteristics. Then, one-dimensional noisy power quality simulation signals are used to estimate the wavelet coefficients through an improved wavelet threshold function. Finally, the denoised motion data is obtained through signal reconstruction. When preprocessing motion data, the collected motion data is first decomposed into multiple levels using wavelet transform to obtain approximation coefficients and detail coefficients at different levels. The calculation is shown in Equation (11).


$$p_{j,k}=\int n\left(t\right)\Psi_{j,k}\left(t\right)dt$$ (11)

In Equation (11), $p_{j,k}$ represents the wavelet coefficient. $\Psi_{j,k}\left(t\right)$ represents the wavelet function. n(t) represents the original signal. Choosing the appropriate threshold is crucial for denoising. The threshold can be based on noise estimation, usually using soft or hard thresholding methods. The threshold λ is shown in Equation (12).


$$\lambda =\sigma \sqrt{2\log N}$$ (12)

In Equation (12), σ represents the noise standard deviation. N represents the signal length. After determining the threshold, the WDC is subjected to threshold processing to preserve the main features of the signal and remove noise components. Finally, the denoised wavelet coefficients are used to reconstruct the signal and obtain the denoised motion data. The denoised motion data is shown in Equation (13).


$$y=\sum j,kd_{j,k}\Psi_{j,k}\left(t\right)$$ (13)

In Equation (13), y represents the denoised motion data. $d_{j,k}$ represents the denoised wavelet coefficients. Assuming that human joints move freely in any way, the collected dataset W(j) is shown in Equation (14).


$$W(j)=\{a_{1}\left(t_{j}\right),a_{2}\left(t_{j}\right),g_{1}\left(t_{j}\right),g_{2}\left(t_{j}\right),{\dot{g}}_{1}\left(t_{j}\right),{\dot{g}}_{2}\left(t_{j}\right\}$$ (14)

In Equation (14), $a_{1}\left(t_{j}\right)$ and $a_{2}\left(t_{j}\right)$ represent accelerations in two different axial directions. $g_{1}\left(t_{j}\right)$ and $g_{1}\left(t_{j}\right)$ represent angular velocities in two different axes. ${\dot{g}}_{1}\left(t_{j}\right)$ and ${\dot{g}}_{2}\left(t_{j}\right)$ are the values obtained by taking the derivative of $g_{1}\left(t_{j}\right)$ and $g_{1}\left(t_{j}\right)$. The time for collecting two sets of data should be a multiple of the sampling period, and these datasets will be applied to determine the unit vectors $j_{1}$ and $j_{2}$ of two IMUs installed on the limbs in the local coordinate system. These unit vectors are constant and their directions are completely determined by the IMU’s installation position relative to the joints. Although $g_{1}\left(t\right)$ and $g_{2}\left(t\right)$ may differ numerically, their projected lengths on the plane defined by the joint axis remain consistent at all times, and the kinematic constraint 0∀t at this time is shown in Equation (15).


$$0\forall t=∥g_{1}(ttimesj_{1}{∥}_{2}-∥g_{2}(t)\times j_{2}∥2$$ (15)

In Equation (15), $∥\cdot {∥}_{2}$ is the Euclidean norm. Regardless of the specific installation position and direction of the IMU on the limb, this kinematic constraint principle always maintains its applicability, and every data acquisition must strictly adhere to this constraint condition. After successfully obtaining the unit vectors $j_{1}$ and $j_{2}$ of the joint axis, the next step is to calibrate the specific position of the IMU relative to the joint center. The fixed vectors $o_{1}$ and $o_{2}$ from the joint center point o to the two IMU positions remain constant throughout the entire process of limb motion, and their values are completely determined by the installation position and direction of the IMU. To ensure data consistency, the acceleration of joint center point o should be consistent in the local coordinate systems of the two IMUs. The kinematic constraint at this point is shown in Equation (16).


$$0\forall t=\left\|a_{1}(t)-\Gamma_{g_{1}(t)}{\left(o_{1}\right)}_{2}\right\|-\left\|a_{2}(t)-\Gamma_{g_{2}(t)}{\left(o_{2}\right)}_{2}\right\|$$ (16)

**Fig 8:**
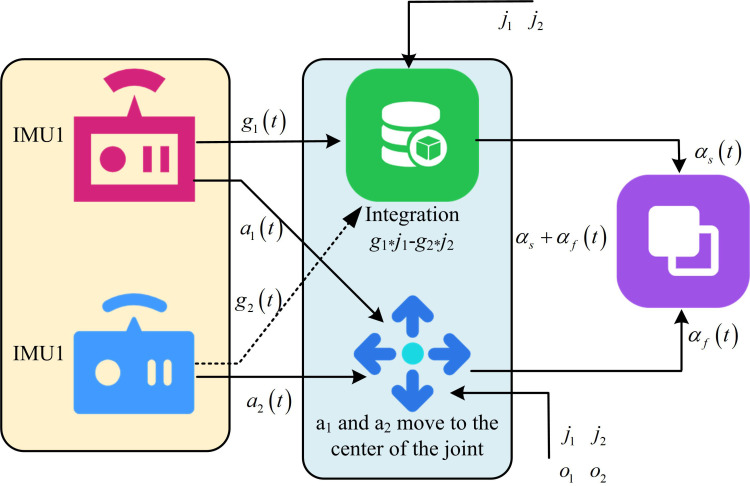
Joint angle calculation.

In Equation (16), Γ signifies the radial and tangential acceleration when rotating around o. After determining the unit vectors $j_{1}$ and $j_{2}$ of the joint axis in the local coordinate system, as well as the $o_{1}$ and $o_{2}$ from the o to the two IMU positions, the joint angle can be calculated. The joint angle calculation is shown in Fig 8.

As shown in Fig 8, the calculation of joint angle is as follows. Firstly, IMU1 and IMU2 are installed in two different parts of the limb, and their respective accelerations $a_{1}\left(t\right)$ and $a_{2}\left(t\right)$, as well as angular velocities $g_{1}\left(t\right)$ and $g_{2}\left(t\right)$, are measured. Secondly, the rotational dynamics of the joint are determined by integrating the angular velocity difference measured by two IMUs, and the joint angle is obtained through the obtained angular velocity difference and time integration. In the process of determining the joint Angle based on the integral angular velocity difference, with the extension of the motion time, the integral will gradually accumulate the small angular velocity errors, resulting in the drift of the Angle measurement. In order to reduce drift, Kalman filter can be used to correct the diagonal speed of other sensor data such as accelerometer in real time to suppress the accumulation of noise and error. The rotation matrix plays a key role in converting the accelerometer data to the joint center coordinate system. By defining the direction and position relationship between different coordinate systems, it maps the measured values of the accelerometer in its own coordinate system to the joint center coordinate system, so as to achieve the consistent expression of data. However, the accuracy of the rotation matrix depends on the prior knowledge of the sensor installation position and orientation, and if there is a deviation in the installation or an error in the sensor itself, the rotation matrix will be inaccurate. To obtain more accurate joint center acceleration, the acceleration measured by the two IMUs is converted into the joint center coordinate system through a rotation matrix. Finally, the final joint angle $\alpha_{s}+\alpha_{f}\left(t\right)$ is obtained by fusing the joint angle $\alpha_{s}\left(t\right)$ calculated by the accelerometer with the joint angle $\alpha_{f}\left(t\right)$ calculated by the gyroscope. The joint Angle calculation algorithm based on IMU can be formalized as follows: Let the output triaxial acceleration of IMU be $a={\left[a_{x},a_{y},a_{z}\right]}^{T}$, the angular velocity be $\omega ={\left[a_{x},a_{y},a_{z}\right]}^{T}$, and the sampling period be Δt. At rest, the initial attitude Angle is calculated by the gravity component of the accelerometer. For details, see Equation (17).


$$\begin{array}{c} \left\{ \begin{array}{c} \theta_{acc}=\arctan\frac{a_{y}}{\sqrt{a_{x}^{2}+a_{z}^{2}}}\;\\ \phi_{acc}=\arctan\;\frac{-a_{x}}{a_{z}} \end{array}\right. \end{array}$$ (17)

The initial Angle of the gyroscope is $\theta_{\text{gyro }}(0)=\theta_{\text{acc, }}\phi_{\text{gyro }}(0)=\phi_{\text{acc }}$. See Equation (18) for the change of gyroscope integral calculation Angle.


$$\begin{array}{c} \left\{ \begin{array}{c} \theta_{\text{gyro }}(t+\Delta t)=\theta_{\text{gyro }}(t)+\omega_{y}\Delta t\\ \phi_{\text{gyro }}(t+\Delta t)=\phi_{\text{gyro }}(t)+\omega_{x}\Delta t \end{array}\right. \end{array}$$ (18)

The accelerometer and gyroscope data are fused by complementary filtering, as shown in Equation (19).


$$\begin{array}{c} \left\{ \begin{array}{c} \theta (t)=\alpha \left(\theta_{\text{gyro }}\right)+(1-\alpha )\theta_{\text{acc }}\\ \phi (t)=\alpha \left(\phi_{\text{gyro }}\right)+(1-\alpha )\phi_{acc} \end{array}\right. \end{array}$$ (19)

In Equation (19), α∈(0,1) represents the fusion coefficient. The adjacent segment IMU coordinate system transformation matrix $R_{j}^{i}=R_{Z}(\psi )R_{Y}(\theta )R_{X}(\phi )$ is defined. The joint rotation constraint equation is established as shown in Equation (20).


$$\min_{q}{\left\|R_{IMU1}\cdot a_{1}-R_{IMU2}\cdot a_{2}\right\|}^{2}$$ (20)

In Equation (20), $q={\left[\psi ,\theta ,\phi \right]}^{T}$ is a quaternion attitude parameter. The relative rotation matrix $R_{rel}=U\cdot V^{T}$ is solved by singular value decomposition (SVD). At each time step t, the current joint Angle is output, as shown in Equation (21).


$$\theta_{\text{joint }}=\arctan\frac{R_{\text{rel }}(3,2)}{R_{\text{rel }}(3,3)}$$ (21)

Through multi-source data fusion and kinematic constraints, sub-degree accuracy (MAE = 1.23°) of joint Angle calculation is achieved, and the computational complexity is O(n).

## 4. Quality analysis of postoperative human motion assessment in orthopedics

### 4.1. Experimental parameters and environmental settings

**Fig 9:**
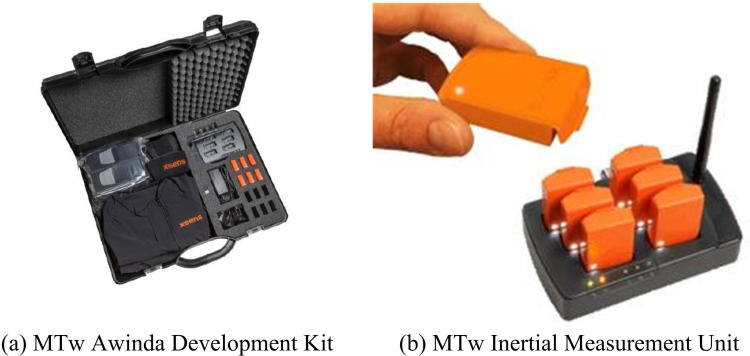
MTw Awinda.

To ensure the universality and robustness of the study results, the study selected 100 participants aged 18–60 years for joint movement data collection experiment. Participants were divided into four age groups (18–25 years, 26–35 years, 36–45 years, 46–60 years), ensuring that 50% of the participants were male and 50% were female. Before the experiment, the *p*-value-values of Body Mass Index (BMI), health status and exercise level of the participants were all greater than 0.05, which was comparable. The participants are in good physical condition, with no abnormalities, have a full understanding of the experimental process, and voluntarily participate. MTw Awinda’s software support and development tools are complete, with a high-precision three-axis accelerometer and gyroscope to provide real-time, high-frequency, low-noise motion data, suitable for dynamic monitoring of the three-dimensional movement of human joints. To ensure the high accuracy and stability of the collected raw data, the MTw Awinda launched by Xsens company is selected as the IMU for the study. A complete MTw Awinda includes one Awinda control station and wireless receiver, six MTw micro IMUs, and other components. The IMU used in the research is shown in Fig 9.

The hardware configuration selected for the experiment is as follows: Windows 10 operating system, 32GB of RAM, 16GB of VRAM, AMD Ryzen 9 5900X @ 3.70GHz CPU, and NVIDIA GeForce RTX 3080 Ti GPU. The selected software is MATLAB R2021a, and Visual Studio 2019. The detailed settings are displayed in Table 1.

**Table 1:** Sample parameter settings.

Image denoising			Multi-scale decomposition of signals		
Parameter Name	Parameter values	Describe	Parameter Name	Parameter values	Describe
Filter strength	0.15	Adjustment of denoising intensity	Decomposition mode	Symmetric	Boundary Processing Mode of Wavelet Transform
Similar block size	7 × 7	Define the size of each pixel neighborhood	Wavelet type	Daubechies 4	The type of wavelet used
Noise level estimated at	10%	Assumed noise level	Signal length	1024	The length of the original signal needs to be filled to 1024
Search window size	21x21	Search for the window range of similar blocks for each pixel	Decomposition level	5	The number of layers in wavelet decomposition

### 4.2. Quality analysis of human joint range of motion measurement after orthopedic surgery

**Fig 10:**
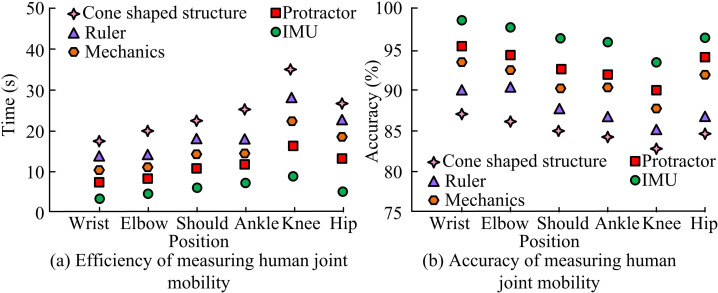
Efficiency and accuracy of measuring human joint range of motion after orthopedic surgery based on IMU.

The measured efficiency and accuracy were compared with other methods under different measurement locations, as presented in Fig 10. In Fig 10 (a), the efficiency of measuring human joint range of motion after orthopedic surgery based on IMU was the lowest for cone measurement method. The reason for this is that cone measurement method requires measuring multiple cross-sections of the cone to obtain comprehensive geometric parameters, which increases the measurement complexity and prolongs the measurement time. At this time, the shortest time for measuring the human wrist joint was 4 seconds and 18 seconds, respectively. In Fig 10 (b), the accuracy of measuring human joint range of motion after orthopedic surgery based on IMU was the highest, with a maximum accuracy of 98% when measuring the human wrist joint.

The average error and standard deviation based on IMU were compared with those of other methods, as displayed in Table 2. In Table 2, IMU had the best performance. The average error and standard deviation of measuring the human wrist joint were the smallest, with an average error and standard deviation of 1.23° and 1.63°. The performance of measuring human joint range of motion after orthopedic surgery based on mechanical measurement was the worst, because mechanical measurement requires operators to have certain skills and experience. If the operator fails to maintain the correct posture or apply uneven force during the measurement process, it will lead to inaccurate measurement results and increase errors. At this time, the average error and standard deviation of measuring the human wrist joint were 2.97° and 3.44°, respectively.

**Table 2:** Performance comparison of human joint range of motion measurement based on IMU after orthopedic surgery.

Position	Average error (°)			Standard deviation (°)		
IMU measurement	Mechanical measurement	Ultrasonic measurement	IMU measurement	Mechanical measurement	Ultrasonic measurement
Wrist joints	**1.23**	2.97	**1.54**	**1.63**	**3.44**	**2.32**
Elbow joint	1.45	3.11	2.17	1.86	3.66	2.55
Shoulder joint	1.77	3.43	2.44	2.02	3.87	2.78
Ankle joint	2.56	**2.75**	3.86	2.57	4.04	3.05
Knee joint	2.62	3.57	3.05	3.11	4.23	3.41
Hip joint	1.67	3.21	2.32	2.46	3.97	2.89

**Fig 11:**
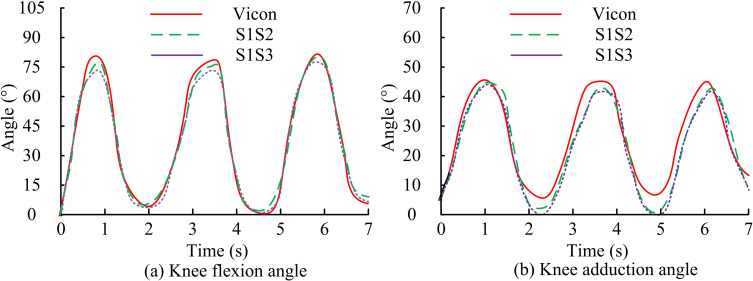
The result chart of measuring the angle of human knee joint motion based on IMU.

The results of measuring the angle of human knee joint motion based on IMU are shown in Fig 11. Among them, the IMU sensor is placed as follows, with an IMU placed on the outer thigh near the knee, denoted as S1. An IMU is placed on the outer side of the calf near the knee, denoted as S2. An IMU is placed in front of the knee, denoted as S3. In Fig 11 (a), in the knee flexion angle, the human knee joint motion angle measured by the proposed method was basically consistent with the results measured by the Vicon optical motion capture system, with a maximum error of 4.9% at 0.8 seconds. In Fig 11 (b), in the knee adduction angle, the results of the human knee joint motion angle measured by the proposed method were basically consistent with those measured by the Vicon optical motion capture system. At 0.8 seconds, the maximum error was 3.7%. The error of the proposed method was less than 5%, which verified the effectiveness of the proposed method.

### 4.3. Quality analysis of human joint angle calculation after orthopedic surgery

**Fig 12:**
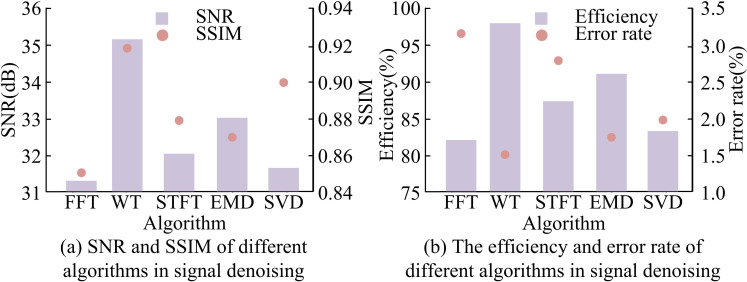
Comparison of denoising performance of IMU acquisition signals using different methods.

The signal-to-noise ratio, visual information fidelity, error rate, and efficiency of different algorithms for multi-scale decomposition of signals collected by IMU are compared, as presented in Fig 12. In Fig 12 (a), the signal-to-noise ratio and visual information fidelity were highest when using Wavelet Transform (WT) to perform multi-scale decomposition on the signals collected by IMU, which were 35.3dB and 0.921, respectively. The signal-to-noise ratio and visual information fidelity of IMU collected signals were the lowest when using Fast Fourier Transform (FFT) for multi-scale decomposition. The reason is that Fourier Transform requires global analysis of the entire signal in time, which has high computational complexity and is not suitable for processing long-term signals. At this time, the signal-to-noise ratio and visual information fidelity were 31.5dB and 0.85, respectively. In Fig 12 (b), compared with other algorithms, the best performance was achieved when WT performed multi-scale decomposition on the signals collected by IMU, with an error rate of 1.52% and an efficiency of 97.4%.

**Fig 13:**
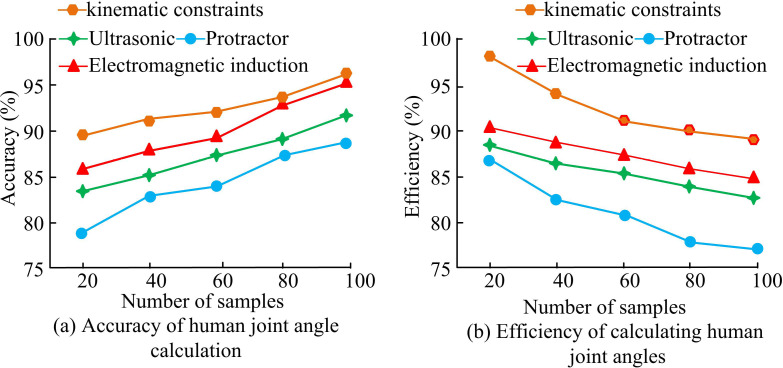
Accuracy and efficiency of different methods for calculating human joint angles after orthopedic surgery.

The accuracy and efficiency of postoperative human joint angle calculation based on kinematic constraints are compared with other methods, as presented in Fig 13. In Fig 13 (a), the accuracy of calculating human joint angles continuously increased with the increase of sample size. The accuracy of calculating human joint angles after orthopedic surgery based on kinematic constraints was the highest, while the accuracy based on protractors was the lowest. When the sample size was 100, the accuracy rates were 93.3% and 86.5%, respectively. In Fig 13 (b), the efficiency of different methods for calculating human joint angles decreased continuously with the increase of sample size. The method proposed in the study had the highest efficiency in calculating human joint angles, with a maximum efficiency of 98.4% when the sample size was 20.

The fitting goodness and maximum error of different methods for calculating human joint angles after orthopedic surgery were compared, as displayed in Table 3. The performance of calculating human joint angles after orthopedic surgery based on kinematic constraints was the best, with the best fitting degree and the smallest maximum error in calculating human joint angles. The fitting goodness and maximum error for calculating joint adduction angle were 0.89 and 1.53°, respectively. The calculated human joint angles after orthopedic surgery on the basis of mechanical measurement was the worst, with a fitting goodness and maximum error of 0.76 and 3.24°, respectively, when calculating joint adduction angles.

**Table 3:** Performance of different methods for calculating human joint angles.

Method	Joint adduction		Joint flexion		Joint adduction and external rotation	
Goodness of fit	Maximum Error (°)	Goodness of fit	Maximum Error (°)	Goodness of fit	Maximum Error (°)
Kinematic constraint method	**0.89**	**1.53**	**0.90**	**3.01**	**0.93**	**1.03**
Traditional protractor measurement	0.79	2.38	0.81	3.96	0.78	2.39
Mechanical measurement	0.76	3.24	0.79	4.07	0.75	2.73
Ultrasonic measurement	0.81	2.04	0.85	3.58	0.84	1.69
Electromagnetic induction measurement	0.86	1.95	0.87	3.23	0.89	1.26

### 4.4 Experimental analysis of potential errors in IMU measurement

In this experiment, 10 healthy volunteers were selected to test the influence of sensor drift and soft tissue artifacts on the measurement of joint Angle by IMU. Experimental group 1 artificially introduced sensor drift, experimental group 2 simulated soft tissue artifacts, and control group was measured normally without any artificially introduced error sources. Independent sample *t* test was used, and the values were expressed in the form of mean ± standard deviation. The effects of sensor drift and soft tissue artifacts on joint Angle measured by IMU are shown in Table 4.

**Table 4:** Test results of potential error sources in IMU measurement.

Experimental condition	Control group	Group 1	Group 2
Joint Angle measurement (°)	30.0, 31.2, 30.5,29.8, 30.3,30.7, 31.0, 30.2,30.6,30.9	32.1, 33.5, 32.8, 31.9, 32.4, 33.0, 32.7, 32.3,33.2,32.6	28.5, 29.0, 28.8,29.2,28.7.29.1,28.9,29.3,29.0,28.6
Mean ± Standard deviation (°)	30.4 ± 0.5	32.7 ± 0.7	28.9 ± 0.4
*t*	/	10.23	-12.45
*p* (compared with control group)	/	<0.001	<0.001

In Table 4, the mean value and standard deviation of experimental group 1 were 32.7° and 0.7°, which significantly increased compared with the control group. The *t* was 10.23, and the *p* < 0.001, indicating that the difference was statistically significant. The mean value and standard deviation of experimental group 2 were 28.9° and 0.4°, which was significantly lower than that of the control group. The *t*-*t*est result was -12.45, and the *p* < 0.001, indicating a significant difference. These results show that both error sources have significant effects on the accuracy of IMU measurement and need to be corrected or compensated in practical applications. Table 5 shows the error suppression effect of different methods.

**Table 5:** Suppression effect of different methods on error.

Method	Drift error (°)	Soft tissue artifact error (°)	*p*-value (vs. raw IMU)
Raw IMU	2.17 ± 0.51	3.81 ± 1.12	<0.0001(drift)
IMU + GPS correction	0.48 ± 0.15	3.65 ± 1.08	<0.0001(artifact)
IMU+ kinematic constraints	1.89 ± 0.43	1.12 ± 0.39	< 0.0001
IMU + GPS+ kinematics constraint	0.32V0.11	0.87 ± 0.28	*p*-value (vs. raw IMU)

In Table 5, the drift error of the original IMU is 2.17° and the soft tissue artifact error is 3.81°. After adding GPS correction, the drift error was significantly reduced to 0.48°, but the soft tissue error was still 3.65°. Combined with kinematic constraints, the drift error is further reduced to 1.89° and the soft tissue error to 1.12°. The IMU + GPS+ kinematic constraint method has the best effect, with drift and soft tissue errors of only 0.32° and 0.87°, respectively, and *p* < 0.0001 compared with the original IMU, indicating that these methods can significantly improve the accuracy of IMU measurement.

## 5. Discussion

In the experimental results, the human joint Angle calculated based on mechanical measurement performed the worst, the goodness of fit of the calculation of joint adduction Angle was only 0.76, and the maximum error was as high as 3.24°. This poor performance is mainly due to inherent flaws in the mechanical measurement method. Mechanical measurement relies on the operator to manually operate the measuring tool to obtain the joint Angle, which is highly dependent on the operator’s skill and experience [31]. If the operator does not accurately place the measuring tool or apply uneven force during the measurement process, it is easy to lead to inaccurate measurement results, which increases the error. In addition, mechanical measurement can not dynamically monitor joint motion in real time, only static or discrete measurement data can be obtained, which is difficult to reflect the continuity and complexity of joint motion [32]. In contrast, methods based on IMU and kinematic constraints have significant advantages. IMU can measure the 3D motion data of joint in real time and continuously, and improve the measurement accuracy through algorithm optimization and data fusion technology. Kinematic constraints further constrain joint motion through mathematical models, reducing errors caused by sensor installation errors or soft tissue interference. These methods combine modern sensor technology and advanced data processing algorithms to more accurately reflect the actual situation of joint motion, and thus perform better in terms of goodness of fit and error control.

## 6. Conclusion

Postoperative human motion assessment in orthopedics can monitor the patient’s rehabilitation progress, provide a basis for adjusting treatment plans, ensure that patients can safely and effectively recover to their optimal functional state, improve their quality of life, and reduce postoperative complications. To improve the efficiency of postoperative human motion evaluation in orthopedics, a method for postoperative human motion evaluation based on kinematic constraints and improved IMU algorithm was proposed. The study first measured the range of motion of human joints after orthopedics surgery based on IMU, and then calculated the joint angles of human joints after orthopedics surgery based on kinematic constraints. The results showed that when the knee joint performed flexion and adduction motions, the method for measuring the human knee joint motion angle was basically consistent with the measurement results of the Vicon optical motion capture system. At 0.8 seconds, the maximum errors were 4.9% and 3.7%, respectively. Based on kinematic constraints, the fitting goodness and minimum maximum error of calculating the joint angle of the human body after orthopedic surgery were achieved. The fitting goodness and maximum error were 0.89 and 1.53°, respectively. The method can effectively measure and calculate the angles of human joints, improving the efficiency of human motion assessment.

## 7. Limitation and future work

Current research methods have made remarkable progress in improving measurement efficiency and accuracy, but there are still some limitations that need to be solved. For example, there is a lack of availability of GPS signals in indoor or complex environments, which directly affects the calibration effect of Inertral Measurement Unit (IMU). In addition, long-term wear of the sensor can lead to dynamic measurement errors, especially those caused by soft tissue displacement. The existing kinematic constraint models have not been able to fully solve the interference of soft tissue artifacts on joint Angle calculation, thus limiting its wide application in clinical practice.

In view of the above problems, future research can be improved from the following aspects. First of all, in order to solve the problem of GPS signals being limited indoors, Ultra Wide Band (UWB) positioning technology can be introduced to build a seamless indoor and outdoor positioning system to ensure that high-precision IMU correction can be achieved in different environments. Secondly, in view of the dynamic measurement deviation caused by long-term wearing of sensors, an adaptive weight allocation mechanism can be introduced to dynamically adjust the kinematic constraint parameters according to the anatomic characteristics of individual patients. At the same time, an intelligent calibration model that does not require a preset installation position is designed to reduce the interference of soft tissue displacement on the measurement results. Through these improvement measures, the feasibility and accuracy of the method in practical application may be further improved.

## Supporting information

S1 file Minimal Data Set Definition. (DOC)
